# Dive surveys and eDNA detect contrasting macroalgal community composition in Nova Scotia, Canada

**DOI:** 10.1111/jpy.70208

**Published:** 2026-07-23

**Authors:** Cody M. Brooks, Claudio DiBacco, Meghan C. McBride, Kira A. Krumhansl

**Affiliations:** ^1^ Department of Fisheries and Oceans Bedford Institute of Oceanography Dartmouth Nova Scotia Canada

**Keywords:** biomonitoring, eDNA, kelp, macroalgae, metabarcoding, non‐indigenous species, qPCR, species composition

## Abstract

Morphological identification of macroalgae can be time‐consuming and species may be difficult to observe, collect, or identify, preventing detailed study on the distribution and ecology of many species. The use of eDNA in species surveillance and biodiversity assessments has gained considerable traction in studying macroalgae, as it provides an opportunity for increased sampling efficiency and a reduced reliance on taxonomic expertise for morphological identification. However, it is crucial to understand how eDNA tools may bias results of species diversity and detection and how these biases differ from traditional morphological surveys. The present study compared species composition and detection rates of dive quadrat collections with a combination of morphological identification and DNA barcoding (i.e., traditional survey methods) to two types of eDNA tools: quantitative polymerase chain reaction (qPCR) using four species‐specific qPCR assays and metabarcoding using two metabarcoding markers. Metabarcoding detected nearly two times more species than traditional surveys; however, species overlap was as low as 19.2%, and some large conspicuous and ecologically important macroalgae were not detected by metabarcoding. This result suggests that metabarcoding uncovers a host of taxa largely distinct from dive surveys. Metabarcoding and qPCR proved capable of detecting target species even at very low abundances, but both showed a high incidence of false negatives. Results presented here support a combined approach leveraging the considerable strengths of eDNA in concert with species validation and abundance metrics provided by tradtional surveys.

**Abbreviations**
BLAST: Basic Local Alignment Search Tool
CEGA: Centre for Environmental Genomics Applications
COI: cytochrome *c* oxidase subunit I
eDNA: environmental DNA
NIS: non‐indigenous species
NMDS: non‐metric multidimensional scaling
OTU: operational taxonomic unit
PERMANOVA: permutational analysis of variance
qPCR: quantitative polymerase chain reaction

## INTRODUCTION

Macroalgae are major components of marine ecosystems on the planet and are presently experiencing rapid global change from shifting environmental conditions (Krumhansl et al., 2016). Most marine ecosystems benefit from macroalgae in several ways, including as sources of primary production that support higher trophic levels, habitat structure to a diverse suite of other organisms (Veiga et al., 2014), coastal erosion mitigation (Innocenti et al., 2018), and blue carbon sequestration (Krause‐Jensen & Duarte, 2018).Macroalgal communities are foundational to oceanic habitats and under threat by species invasions, urbanization and rapid warming (Garcia et al., 2020; Gabara et al., 2021; Hargrave et al., 2017; Hildebrand et al., 2024; Saunders & Metaxas, 2008; Wilson et al., 2015), yet in many regions the composition and diversity of macroalgal communities remains poorly understood.

Documenting species composition with traditional macroalgal sampling methods is complicated by the existence of heteromorphic life histories, simple or plastic morphologies, an abundance of cryptic species, and high phylogenetic diversity (Kucera & Saunders, 2012; Vieira et al., 2024). Traditional methods of collecting and surveying macroalgae include drop camera surveys (Vandermeulen, 2018), dive surveys (Smale et al., 2011), or dredging (Husa et al., 2008). The taxonomic expertise required to identify these (and other) organisms morphologically has been on a global decline for some time (Hopkins & Freckleton, 2002), and as such, macroalgae are often classified only at coarse taxonomic levels (e.g., as turf or kelp in Filbee‐Dexter et al., 2016). The accurate morphological identification of species can be time‐consuming and expensive, but misidentifications can affect ecological interpretation when cryptic species have differing physiological limits (Dankworth et al., 2020; Vieira et al., 2023). DNA barcoding has been used in concert with morphological identification to confirm species identifications and elucidate cryptic complexes (Saunders et al., 2025), but this adds further time and cost to biodiversity assessments in order to preserve, amplify, and generate sequence data for collections.

Molecular tools provide a powerful alternative to traditional morphological methods for characterizing macroalgal diversity and distributions (Borer et al., 2025). Environmental DNA (eDNA) technologies involve the extraction, isolation, and identification of extracellular DNA from an environmental sample (e.g., water, air, or soil), which can be used in a variety of applications. In recent years, eDNA methods have been used to great effect in marine surveillance to study biodiversity (Kelly et al., 2018), monitor for non‐indigenous species (LeBlanc et al., 2020), and document rare or endangered species (Harper et al., 2018). Environmental DNA methods have some advantages over traditional sampling, as they are non‐invasive, more sensitive, and detect higher species richness (Borer et al., 2025; Gold et al., 2021; Jeunen et al., 2022; Ruppert et al., 2019). Further, eDNA sampling is generally more efficient, allowing for broader spatial and temporal coverage than traditional methods. Conversely, eDNA metabarcoding is costly to implement, requires expertise in bioinformatics, and is limited by genetic reference databases and detections that do not always match traditional sampling for various reasons (discussed below).

The present study addressed two types of eDNA methods for macroalgal sampling: quantitative polymerase chain reaction (qPCR) and metabarcoding. In qPCR, a polymer is amplified from an eDNA sample, and the fluorescence of a reporter molecule is measured during each PCR amplification cycle allowing reasonably accurate measurements of DNA quantities in the initial sample (VanGuilder et al., 2008). In qPCR, assays usually target a single species, and a separate assay must be developed for every species individually. Quantitative PCR is a relatively mature technology in marine biosurveillance, and many species‐specific assays are already available, although assays for macroalgae are relatively uncommon compared to those for fish and invertebrate species (LeBlanc et al., 2020). For detecting one or a few species, qPCR is more cost‐effective than metabarcoding (Lafferty, 2024) if assuming a new qPCR assay does not need to be designed. Moreover, technology is now available for real‐time, in situ, and autonomous qPCR for detecting and quantifying species, making qPCR a powerful tool for rapid marine biomonitoring (Preston et al., 2011). By contrast, metabarcoding aims to generate unique DNA sequences (i.e., barcodes) for every species present in an eDNA sample, which is achieved using universal primers to amplify a short, conserved marker region, typically a fragment of the 18S rRNA or COI gene. Each sample is multiplexed (i.e., pooled into a single reaction) and sequenced on a high‐throughput platform, producing millions of individual reads. These sequences are then processed with a suite of software (i.e., a bioinformatics pipeline) that demultiplexes, denoises, filters contaminant sequences, and assigns species names to sequences using a reference database (see Hakimzadeh et al., 2023). Metabarcoding is generally used to detect entire species communities, whereas qPCR targets a specific species with higher precision. Although qPCR has often been reported to be more sensitive than metabarcoding for species‐specific detection (Wood et al., 2019), sensitivity depends on the species, primers, and target gene. Notably, metabarcoding studies increasingly employ multiple markers, which may enhance species detection rates.

Metabarcoding is increasingly used in favor of traditional tools to assess species diversity and composition, yet metabarcoding does not necessarily detect the same species as traditional methods. Species physical traits can influence their detection with metabarcoding; for example, invertebrate species with hard exoskeletons can be more difficult to detect with eDNA than those with soft bodies (Lancaster et al., 2025). For macroalgae, robust morphologies (e.g., *Agarum clathratum*) may shed less material than those with more delicate forms (e.g., *Bonnemaisonia hamifera*), which may skew eDNA detections. Species detections with metabarcoding are also strongly influenced by the primer set used, which is complicated for macroalgae because they have broad phylogenetic diversity. Due to this diversity, species barcoding has necessitated the development of innumerable primers for red algae alone (Saunders & Moore, 2013). Also, mismatches between eDNA and traditional methods can occur because the latter are known to overlook small, rare, ephemeral, or morphologically cryptic species. A recent review of 215 datasets confirmed that eDNA tools often do not capture the same taxa as traditional methods (Keck et al., 2022), but this review did not include marine macroalgal studies. The alignment between metabarcoding methods and traditional tools for surveying macroalgal diversity remains largely unknown.

To address gaps in our understanding of how eDNA tools can be used to assess macroalgal species distributions and community composition, we employed a set of eDNA methods in combination with traditional sampling (dive surveys with morphological identification and DNA barcoding) with the following objectives: (1) to compare species assemblages identified through eDNA metabarcoding with those observed using traditional surveys for macroalgae and (2) to compare species detection rates from four species‐specific qPCR assays against the detection rates obtained using two general metabarcoding primers, validated with traditional surveys. Since most metabarcoding studies recommended using at least two markers, we also evaluated the combined sensitivity of the two metabarcoding markers relative to each of the four qPCR assays for targeted species detection.

## MATERIALS AND METHODS

### Sampling sites

This study applied two sampling methods, dive surveys and eDNA collection, across six sites along the Atlantic coast of Nova Scotia occurring within ~500 m from shore (Figure 1, Table 1). Sampling typically occurred between July and September, with both methods conducted on the same day or within 2–3 days of each other. Exceptions included Gill Cove and Sober Island, where eDNA samples were collected in 2022 approximately 2 months earlier (Gill Cove) and 1 month earlier (Sober Island) than the corresponding dive surveys. Beaver Point and Crystal Crescent were sampled in 2021. Black Island and Charlotte Island were sampled in 2022, and Gill Cove and Sober Island were sampled in both 2021 and 2022. In total, there were eight sampling events.

**FIGURE 1:**
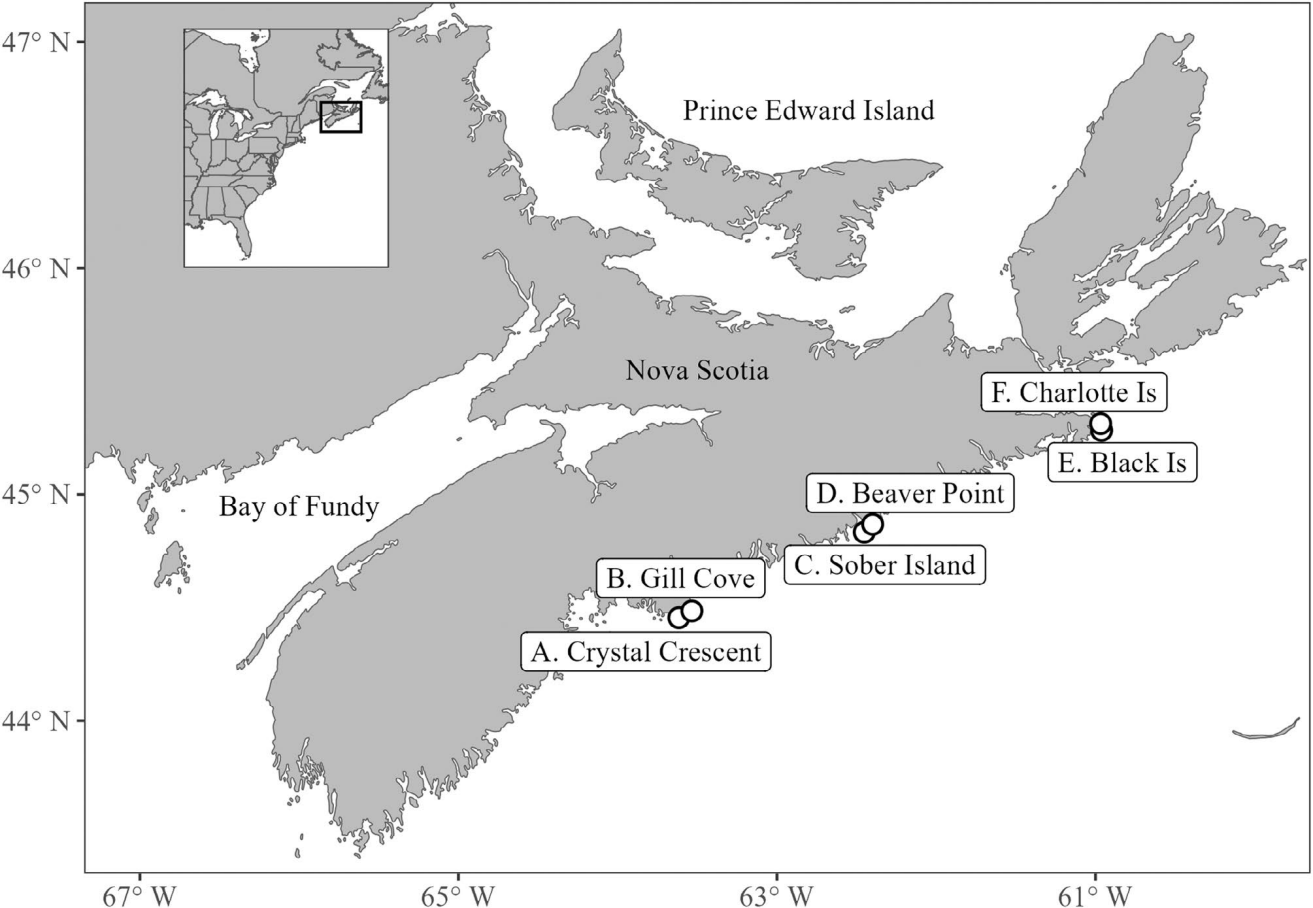
Study sites in this study included A. Crystal Crescent, B. Gill Cove, C. Sober Island, D. Beaver Point, E. Black Island, and F. Charlotte Island.

**TABLE 1:** Sites sampled with qPCR, metabarcoding and dive surveys in this study, including the number of samples of each type collected.

Site	Year	Latitude	Longitude	Dives	eDNA	
					Metabarcoding	qPCR
A. Crystal Crescent	2021	44.455	−63.616	16	4	8
B. Gill Cove	2021	44.485	−63.534	16	6	6
B. Gill Cove	2022	44.485	−63.534	12	3	3
C. Sober Island	2021	44.825	−62.445	16	3	3
C. Sober Island	2022	44.825	−62.445	12	3	3
D. Beaver Point	2021	44.868	−62.400	16	3	3
E. Black Island	2022	45.357	−61.142	12	3	3
F. Charlotte Island	2022	45.312	−60.968	12	3	3

*Note*: All field sampling was conducted between July and September.

### Dive surveys

Dive surveys were performed by SCUBA from a boat with six to eight quadrats spaced 2–3 m apart, sampled at each of two depth ranges per site (i.e., deep = 8–10 m; shallow = 3–5 m mean lower low water, MLLW). Sampling therefore covered a 12–15‐m transect per depth per site, representing a logistically feasible sampling protocol for one dive per site. For each sample, a 0.75‐m^2^ quadrat was haphazardly placed on the benthos, and canopy species (e.g., kelps, fucoids) were enumerated and removed from the substratum. Additionally, after removing canopy species, all remaining visible macroalgae within a 0.25‐m^2^ subquadrat were collected using a scraper, retained in a 1‐mm mesh bag, stored on ice, and returned to the Bedford Institute of Oceanography (BIO) for identification. Cobbles and other substrates that may support crustose algae were not collected. This survey method was used over a roving diver approach, as it enabled quantification of species abundances rather than just occurrence and is a common method used in subtidal monitoring programs (kelpecosystems.org).

In the lab, subquadrat species were sorted by gross morphology and microscopy and identified following Saunders (2024) and Mathieson and Dawes (2017). In 2021, DNA extraction, PCR amplification, and sequencing were performed for 109 samples to confirm selected morphological identifications. Only red algal species were sequenced because these were the most common and are the most difficult to identify morphologically. For each sample to be sequenced, a 1‐cm^2^ section of frozen material was desiccated in silica, extracted and amplified following Saunders and Moore (2013) at the Center for Environmental and Molecular Algal Research (CEMAR) at the University of New Brunswick (Fredericton, New Brunswick, Canada). Successful DNA extracts of red algae were amplified using forward primer F57 (Freshwater & Rueness, 1994) and reverse primer rbcLrevNEW (Saunders & Moore, 2013) to target the 3′ end of the *rbc*L gene region. All successful amplicons were sequenced using their respective reverse primers at Génome Québec on an Applied Biosystems 3730xl DNA Analyzer. Raw data were imported and edited on Geneious Prime 2020.1.1 (Biomatter Inc., San Diego, California, United States) to a final length of approximately 840 bp and compared to sequences available on GenBank and the Barcode of Life Database (BOLD) to confirm species identification. Informal species (e.g., *Polysiphonia* sp. 3 stricta) were sunk into generics (e.g., *Polysiphonia* spp.).

### Environmental DNA water collection

At each sampling site, three eDNA samples and one field blank were collected. For each sample, sterilized 1‐L amber bottles were triple‐rinsed with ambient seawater and subsequently filled with seawater collected from ~1 m above the bottom, either using a well pump or by SCUBA. A fourth bottle prefilled with distilled water was manipulated at each site (opened, partially poured out, and then resealed) to serve as a field blank. All bottles were transported on ice to the laboratory. Samples were either stored in a refrigerator and processed within 24 h or frozen (−20°C) and processed at a later time. In total, 32 samples, including field blanks, were filtered using Sterivex filters (0.22 μm) and preserved with Longmire buffer. Filters were stored at −80°C until DNA extraction.

### Environmental DNA extractions and quantitative polymerase chain reaction

DNA extractions were performed on all 32 samples collected in 2021 and 2022, including field blanks, from six sites across Nova Scotia. Extractions were conducted by the Centre for Environmental Genomics Applications (CEGA, St. John's, Newfoundland, Canada) using the DNeasy PowerWater Kit (Qiagen) followed by DNA quantification. Extraction negatives were included as part of quality control to monitor for contamination. In 2021, CEGA developed and applied three species‐specific qPCR assays targeting *Bonnemaisonia hamifera*, *Dasysiphonia japonica*, and *Laminaria digitata* (Table S1) to 16 samples collected from Beaver Point, Crystal Crescent, Gill Cove, and Sober Island. Each 20‐μL qPCR reaction contained 1× LightCycler 480 Probes Master, 300 nM of each primer, 150 nM of probe, and 1 μL of template DNA. Reactions were run in triplicate under the following cycling conditions: initial denaturation at 95°C for 10 min, followed by 55 cycles of 95°C for 15 s and 60°C for 60 s.

For species‐specific detection in 2022, 16 samples from Charlotte Island, Black Island, Gill Cove, and Sober Island were analyzed in the Aquatic Biotechnology Lab (ABL) at the Bedford Institute of Oceanography (BIO) within designated eDNA clean labs. The qPCR assays targeting *Bonnemaisonia hamifera* and *Dasysiphonia japonica* followed a protocol similar to CEGA's, with each 20 μL containing 300 nM of each primer and 150 nM of probe, 1× TaqPath ProAmp Multiplex Master Mix, and 3 μL of template DNA. Thermocycling conditions matched those used by CEGA. For 2022 samples, a newly developed and validated assay was applied for *Antithamnionsparsum* following the local occurence of this species (Brooks & Krumhansl, 2023). The assay for *Laminaria digitata* was not used in 2022. Each qPCR reaction for *A. sparsum* included 1× TaqPath ProAmp Multiplex Master Mix, 800 nM of each primer, 75 nM of probe, and 3 μL of template DNA in a total volume of 20 μL. Amplification was performed in triplicate for each sample using the following thermocycler program: 50°C for 2 min, 90°C for 10 min, 40 cycles of 95°C for 15 s, and 62°C for 60 s. Primer information for all qPCR assays is listed in Table S1.

### Environmental DNA metabarcoding

Metabarcoding libraries for all 2021–2022 samples were prepared at CEGA using two universal primer sets targeting the Leray fragment of the COI gene (Leray et al., 2013) and the V4 region of the 18S rRNA gene (Nelles et al., 1984; Table S2). Polymerase chain reaction amplification and cycling conditions followed published protocols for the COI (Derycke et al., 2021) and 18S rRNA (Stat et al., 2017) genes. Final COI and 18S rRNA gene libraries were sequenced on an Illumina NovaSeq™ 6000 instrument using a 2 × 300 bp kit with a target depth of 1,700,000 reads per sample.

### Bioinformatics pipeline and species list curation

A custom bioinformatics pipeline was developed, originally based on MicrobiomeHelper (Comeau et al., 2017). Semi‐raw reads from CEGA arrived trimmed of adapter and primer sequences. These trimmed, paired‐end reads were merged using NGmerge v.0.3 (Gaspar, 2018) and denoised using DnoisE v.1.4.1 (Antich et al., 2021). Singletons were removed prior to taxonomic assignment by the Basic Local Alignment Search Tool (BLAST) using a local copy of the nr/nt database (downloaded November 2023), and sequences were clustered at 99% similarity using VSEARCH v2.29.3 (Rognes et al., 2016). Following taxonomic assignment, we retained only operational taxonomic units (OTUs) with 10 reads or more in a given sample and which were a good match to a GenBank record (≥99.0% identity for the 18S rRNA gene and ≥97.5% for the COI gene). Non‐macroalgal taxa were removed to facilitate relevant comparisons of richness and biodiversity with macroalgal dive surveys. Retained algal species included those with both macro‐ and microscopic stages (e.g., *Acrosiphonia* spp., *Porphyra* spp.) as well as crustose and intertidal species. The final species table was reviewed for outdated species names incorrectly assigned by BLAST and probable misidentifications resulting from genetic identicality between two or more species across the selected Leray fragment or V4 marker regions. Informal species (e.g., *Polysiphonia* sp. 3stricta) were sunk into generics (e.g., *Polysiphonia* spp.), the same as for DNA barcoding. Following the above quality‐control steps, samples were pooled and species were treated as present if they occurred in at least one eDNA replicate at a site.

After removing low quality reads and those with poor species‐level matches to the GenBank database (<99.0% identity for the 18S rRNA gene and <97.5% identity for the COI gene) and non‐macroalgal taxa, the Leray fragment and 18S–V4 markers returned 2.12 million and 3.86 million paired‐end reads, respectively, from 24 samples. The data for both markers were combined for subsequent analysis.

### Data analysis

Data from all methods was imported into R (version 4.3.2; R Core Team, 2023). The similarity of species assemblages was calculated as the percentage of species detected by both metabarcoding and dive surveys at each site. Species detected by dive surveys but not eDNA were given one of the following reasons: (i) no amplification: species with available reference sequences (those without available reference sequences were excluded) that were not detected by eDNA, potentially due to poor primer fit or no/low DNA concentration. Species detected by eDNA but not by dive surveys were categorized as follows: (ii) offsite: eDNA from intertidal or estuarine species not present or expected at subtidal study sites; (iii) cryptic: morphologically cryptic species identifiable only through molecular methods; (iv) ephemeral: macroalgae with alternating microscopic and macroscopic life stages, wherein the macroscopic stage was absent during dive surveys; (v) minute: species <5 cm at maturity and not abundant, potentially overlooked during dive surveys; and (vi) crustose: species with crustose gross morphology, which were not collected during dive surveys in this study. Finally, species that could not be classified under the above categories were assigned as (vii) unknown.

The detection rate was calculated as the number of eDNA samples with positive detections from sites where a given species was also observed during dive surveys. A failure to detect a given species using either qPCR or metabarcoding was considered a false negative if that species was present in dive surveys. Positive detections were compared between eDNA methods (qPCR and metabarcoding) using a generalized linear mixed effects model (GLMM) implemented with the glmer function in the lme4 package in R (Bates et al., 2015). The model accounted for the random effects of site (Beaver Point, Crystal Crescent, Gill Cove, Sober Island, Black Island, and Charlotte Island) and species (*Anithamnion sparsum*, *Bonnemaisonia hamifera*, *Dasysiphonia japonica*, and *Laminaria digitata*) but not year, as this factor did not account for significant variation in the model. Response data were coded as 0 (detected only by dive surveys) or 1 (detected by eDNA and dive surveys) for each eDNA method (qPCR and metabarcoding) across site, species, and sampling year. As such, the data were modeled using a binomial error distribution. Prior to analysis, data were assessed for overdispersion, underdispersion, and homogeneity of variances using the simulateResiduals function in the DHARMa package in R (Hartig, 2024).

To compare species diversity captured by metabarcoding and dive surveys, we used non‐metric multidimensional scaling (NMDS) implemented with metaMDS from the vegan package v2.6‐10 (Oksanen et al., 2025). The NMDS was performed on the community assemblages using Jaccard distances calculated from the matrix of presence and absence data, repeating 50 iterations until the lowest stress for *k* = 2 dimensions was achieved. A permutational analysis of variance (PERMANOVA) with 9999 permutations was conducted to identify significant differences among site, year, and method using adonis2 (vegan package). Spearman's ρ correlations were used to assess relationships between a sampling event's NMDS1 value and the number of species at a site within biotic categories, including depth (subtidal and intertidal), morphology (crustose, fine turf, coarse turf, canopy, and minute), and cryptic status.

## RESULTS

### Species diversity

Canopy species from dive surveys were predominantly *Laminaria digitata* and *Saccharina latissima* except at Charlotte Island and Black Island, where the non‐indigenous macroalga *Fucus serratus* dominated by biomass (Table 2). Turf species recovered in dive surveys were dominated by six species of the family Gigartinales (i.e., *Chondrus crispus*, *Coccotylus truncatus*, *Cystoclonium purpureum*, *Furcellaria furcellata*, *Phyllophora pseudoceranoides*, and *Polyides rotunda*) comprising 56.2% of turf biomass (Table 2). Metabarcoding samples were overwhelmingly dominated by *Dasysiphonia japonica*, representing 86.4% of Leray fragment reads post‐filter (3.7 million reads) and 84.4% of 18S–V4 reads post‐filter (6.5 million reads; Table S3). Of the five kelp species (order Laminariales) present in this flora, only *L. digitata* was identified by metabarcoding and only with the Leray fragment (Table S3).

**TABLE 2:** The percentage of water samples with positive detections from sites where a species was observed during dive surveys.

Species	Metabarcoding detections (%)	Mean biomass (g · 0.25 m^−2^)
*Fucus serratus*	83.3	540.3 ± 491
*Laminaria digitata*	85.7	523.4 ± 347.7
*Corallina officinalis*	89.3	164 ± 186.9
*Coccotylus truncatus*	35.7	105.3 ± 126.7
*Chondrus crispus*	89.3	59.3 ± 74.4
*Phyllophora pseudoceranoides*	22.7	54.5 ± 62.9
*Polyides rotunda*	15.8	48 ± 141.2
*Furcellaria lumbricalis*	16.7	42.6 ± 116.2
*Bonnemaisonia hamifera*	80.0	18.7 ± 23.2
*Antithamnion sparsum*	76.9	15.9 ± 33.6
*Fucus distichus*	42.1	10.1 ± 16.4
*Dasysiphonia japonica*	63.6	8.4 ± 13.8
*Ahnfeltia plicata*	14.3	8.3 ± 16.5
*Cystoclonium purpureum*	60.7	7.7 ± 14.5
*Spermothamnion repens*	5.3	7.2 ± 12.4
*Desmarestia viridis*	87.5	6.5 ± 5.5
*Ptilota serrata*	6.2	5.4 ± 11.7
*Sphaerotrichia divaricata*	100	4.4 ± 3.2
*Ceramium virgatum*	52.0	3.7 ± 6.2
*Vertebrata* spp.	44.0	3.3 ± 3.5
*Phycodrys* spp.	48.0	3 ± 5.1
*Palmaria palmata*	8.3	2.9 ± 3.7
*Euthora cristata*	40.0	2 ± 3.6
*Scytosiphon* spp.	0	1.3 ± 0.2
*Polysiphonia* spp.	73.3	1.2 ± 2.5
*Rhodomela lycopodioides*	22.7	1.1 ± 1.7
*Callithamnion corymbosum*	16.7	0.9 ± 1.7
*Membranoptera fabriciana*	36.8	0.9 ± 1.5
*Antithamnionella floccosa*	33.3	0.7 ± 1
*Dumontia contorta*	0	0.4*
*Wildemania amplissima*	0	0.3*
*Sphacelaria/Sphaceloderma* spp.	0	0.1 ± 0.1
Combined	42.5	

*Note*: Associated mean biomass (g per 0.25 m^2^ dry weight) and standard deviation are provided. Asterisks indicate where only one sample was collected, so there is no standard deviation value.

Dive surveys yielded a cumulative 43 taxa, whereas metabarcoding detected 72 taxa; combined, both methods identified 84 unique taxa spanning 23 orders and 46 families (Table S3; Figure 2a). Dive surveys only uncovered 12 unique taxa (14.3% of species detections) from metabarcoding (Figure 2a); however, this cohort included ecologically significant canopy species (e.g., *Agarum clathratum* and *Saccharina latissima*) and the non‐indigenous species *Codium fragile*. The frequency of positive eDNA detections varied across species: Taxa with high biomass were detected in most samples (e.g., *Chondrus crispus*, 89.3% and *Corallina officinalis*, 89.3%), as were species of moderate biomass with many delicate, easily fragmented branches (e.g., *Bonnemaisonia hamifera*, 80.0% accuracy; *Desmarestia viridis*, 87.5% accuracy; Table 2). In contrast, coarse and robust species that do not fragment easily were rarely detected by metabarcoding, potentially due to low shedding rates (e.g., *Ahnfeltia plicata*, 14.3% accuracy, and *Polyides rotunda*, 15.8% accuracy; Table 2).

**FIGURE 2:**
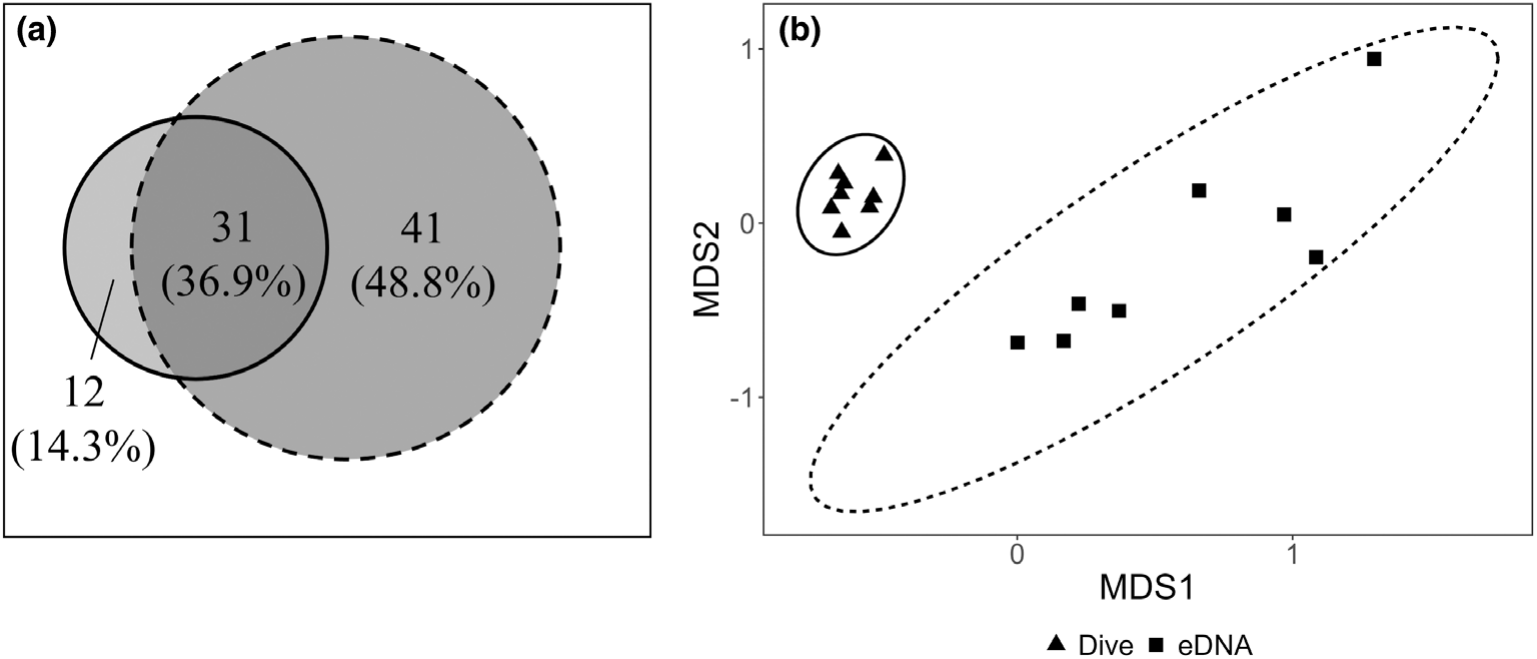
Panel (a) shows total species richness captured by dive surveys only (solid line), metabarcoding only (dashed line), and both methods (overlap), (b) shows results of non‐metric multidimensional scaling (NMDS) performed on Jaccard distance matrixes of community assemblages captured by dive surveys and metabarcoding (2D Stress = 0.096).

The similarity in species detections between dive surveys and metabarcoding ranged from 19.2% to 35.1% across sites, with eDNA detecting more species than dive surveys during five out of eight sampling events (Figure 3). Dive surveys, however, detected more species than eDNA at three sampling events in 2022 (at Sober Island, Black Island, and Charlotte Island; Figure 3). Overall, 22.9% of eDNA‐only species detections originated from intertidal or estuarine taxa (e.g., *Gayralia* sp.), likely introduced from off‐site locations, whereas 25.2% of eDNA‐only detections were species easily overlooked by dive surveys, including 7% that were cryptic, 5.8% that were crustose, 4.7% that were ephemeral, and 2.3% that were minute (Figure 4). Conversely, 14.4% of species observed during dive surveys were not detected by eDNA at any site despite adequate reference sequences in GenBank (Figure 4). These failures are likely the result of primer mismatches, low DNA concentrations, poor DNA quality, or the absence of DNA in the sampled water column.

**FIGURE 3:**
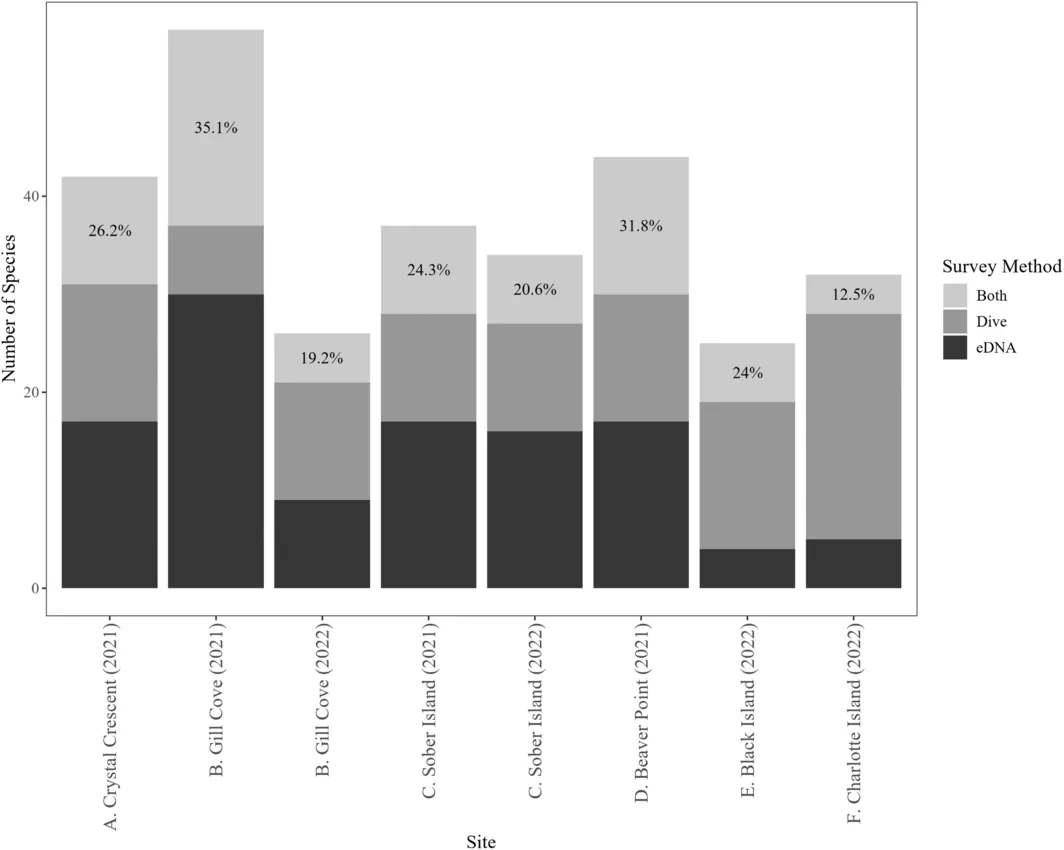
Species richness from dive surveys and metabarcoding, including false negatives (species present in dive surveys but absent from metabarcoding surveys, “Dive”), eDNA‐specific detections (species absent in dive surveys but present in metabarcoding surveys, “eDNA”), and overall similarity of species compositions between dive surveys and metabarcoding (“Both”).

**FIGURE 4:**
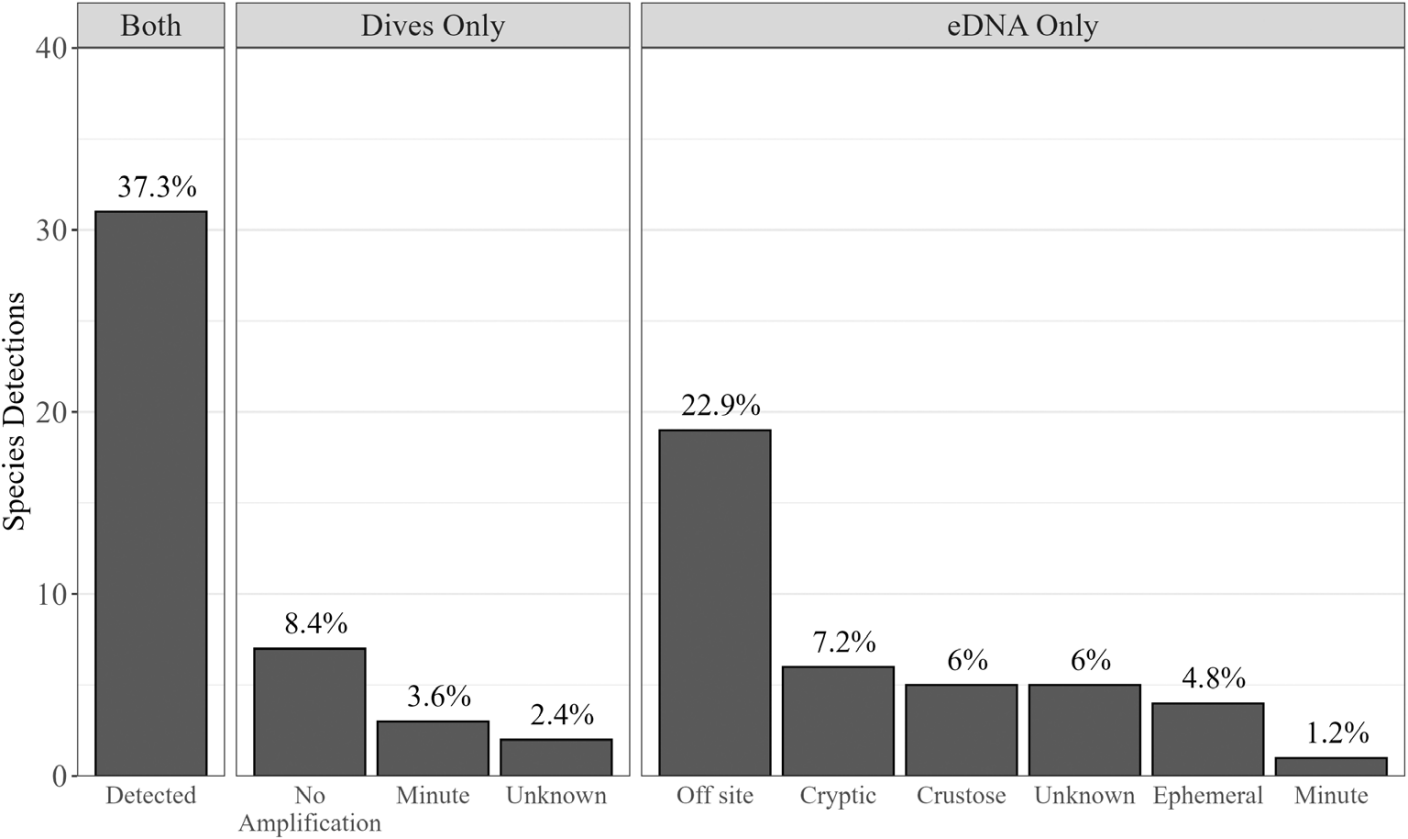
The number of species detections by site classified by method of detection. Classifications are defined as follows: (i) no amplification: Species which were never detected by eDNA but for which adequate reference sequences exist; (ii) minute: Species which are <5 cm at maturity but not microscopic; (iii) off site: Intertidal or estuarine species; (iv) cryptic: Species which are morphologically cryptic; (v) crustose: Species with crustose gross morphology; (vi) ephemeral: Macroalgae which are only present in macroscopic form during a short season, and whose season did not align with the dive surveys; (vii) unknown: Species which could not be classified using the terms above. Percent values above each bar indicate the proportion of all detections assignable to that classification.

The NMDS provided a good 2D visualization of the data (2D stress = 0.096), with sites separating most clearly by method along NMDS1 (Figure 2b). A PERMANOVA confirmed a significant effect of method on species composition (*R* ^2^ = 0.32, *F* = 7.7114, *p* = 0.0001, *df* = 1), but not site (*R* ^2^ = 0.27, *F* = 1.2972, *p* = 0.1499, *df* = 5) or year (*R* ^2^ = 0.06, *F* = 1.3948, *p* = 0.1623, *df* = 1). Environmental DNA metabarcoding samples detected more cryptic (ρ = 0.646, *p* = 0.00682) and crustose (ρ = 0.504, *p* = 0.04637) species than dive surveys, determined by a significant Spearman's correlation between the number of species in each of these categories and sample separation along NMDS1. Conversely, dive surveys detected more non‐cryptic species (ρ = −0.586, *p* = 0.01705) than eDNA metabarcoding. The number of intertidal, subtidal, coarse turf, fine turf, and minute species detected during each sampling event was not correlated with sample separation along NMDS1, indicating no consistent difference between dives and metabarcoding samples in detecting species in these categories (Table S4).

### Detections of target species

The second aim of this study was to assess the ability and consistency of eDNA sampling in detecting target species selected to represent all three macroalgal phyla. Positive detections and false negative rates are reported in Table 3. Across four target species, qPCR returned positive detections in 67.4% of samples, whereas the combined Leray and 18S–V4 markers detected 76.6% of samples. A generalized linear mixed effects model indicated no significant difference between qPCR and metabarcoding in detecting these four target species (Table 4). This result indicates that overall, there is no consistent advantage of one method over the other for target species detection. There was not enough statistical power to test for an interaction between species and method on detection rates, but the efficacy of the two methods appeared to vary by species (Table 3). Detection rates by qPCR were lower than metabarcoding for *Anithamnion sparsum* and *Bonnemaisonia hamifera*, the former by a significant margin. In contrast, detection rates for *Dasysiphonia japonica* and *Laminaria digitata* were higher for qPCR than metabarcoding (Table 3).

**TABLE 3:** The percentage and corresponding number of positive detections (calculated as the number of water samples with a positive detection relative to the total number of samples collected at sites where the species was also detected from dive surveys, in brackets after the dection %) for each eDNA method (qPCR, metabarcoding).

Species	qPCR		Metabarcoding	
	Detections (%)	FN	Detections (%)	FN
*Antithamnion sparsum*	33.0 (1/3)	2	76.9 (10/13)	3
*Bonnemaisonia hamifera*	62.5 (20/32)	9	80.0 (20/25)	5
*Dasysiphonia japonica*	73.9 (17/23)	6	63.6 (14/22)	5
*Laminaria digitata*	100 (20/20)	5	85.7 (24/28)	4
Overall mean	67.4%		76.6%	

*Note*: False negatives (FN) are defined as sites where eDNA failed to detect a species detected in dive surveys. Note that *Anithamnion sparsum* was only targeted by qPCR in 2022, and *Laminaria digitata* was only targeted by qPCR in 2021.

**TABLE 4:** Results of a generalized linear mixed effects model comparing positive detections of target species (*Anithamnion sparsum, Bonnemaisonia hamifera, Dasysiphonia japonica,* and *Laminaria digitata*) between two eDNA methods: qPCR and metabarcoding.

Random effects				
Factor	Variance	*SD*		
Species	2.051	1.432		
Year	1.112	1.055		
Site	1.376	1.173		

Fixed effects				
Factor	Estimate	*SE*	*z*	*p*
eDNA method	0.3812	0.7656	0.498	0.619

*Note*: Shown are the variance and standard deviations (*SD*) associated with the random effects of species and year, and the coefficient estimates (estimate), standard error (*SE*), *z*, and *p*‐value associated with the fixed effects of eDNA method.

## DISCUSSION

We found high dissimilarity in macroalgal species detection and composition between eDNA and dive surveys. While eDNA captured greater macroalgal species diversity, this assemblage was distinct from traditional surveys and overlooked several common, abundant taxa. Conversely, dive surveys missed a significant portion of the diversity, including species that are difficult to sample by quadrats (e.g., crusts, cryptic), those absent due to the seasonal timing of sampling (ephemeral), and those occurring only rarely at a site. Metabarcoding also detected taxa likely originating from nearby intertidal or estuarine habitats rather than locally. For target species detection, eDNA proved efficient for broad‐scale screening; however, inconsistent positive detection rates indicate it cannot be solely relied on to characterize targeted species distributions.

### Species composition

In this and other studies, metabarcoding consistently identified higher species richness (Table 2; Borer et al. 2025; He et al., 2022; Klunder et al., 2022), yet species composition from metabarcoding and dive surveys shared only 36.5% of taxa on average. This aligns with findings by Borer et al. (2025) for macroalgal communities in Portugal, for which species detected by metabarcoding and traditional survey methods (photo quadrats) showed 23% similarity. This is also similar to the findings from Keck et al. (2022) for other species groups, which found metabarcoding and traditional methods detected different suites of species. The discrepancy we observed here is slightly less than the 50% similarity observed in benthic, littoral macroinvertebrates but comparable to diatoms for which only 29% of taxa overlapped between methods, likely reflecting similar challenges in morphological identification (Aylagas et al., 2016; Pérez‐Burillo et al., 2020).

Compositional differences in other species groups have been attributed to a various factors, including seasonal variations in species occurrences (Zhao et al., 2021). Macroalgae exhibit strong seasonal changes in abundance and life‐history stages, which may contribute to differences in species detections between dive surveys and metabarcoding. This may be particularly true for species with alternating macro‐ and microscopic stages (e.g., *Acrosiphonia arcta*), for which the microscopic stage may remain detectable by eDNA but not by traditional methods. In this study, the presence of drifting, “off site” taxa accounted for 23.3% of all eDNA‐only species detections (Figure 4). These taxa were predominantly intertidal or estuarine species, many of which are buoyant (e.g., *Ascophyllum nodosum*, *Fucus vesiculosus*) and likely originated from outside of the area assessed by dive surveys (Figure 4). Drift material was likely amplified, meaning these detections did not represent locally established species within the survey area. Previous studies have shown that for other species groups and systems, eDNA diversity metrics may include detrital and/or drift material from local benthos or intertidal habitats or nearby habitats, (Phillips, 2001). Drifting taxa may travel 10s to 1000s km away from their sources while remaining detectable in eDNA (Ortega et al., 2019) and may shed DNA into the water column during transport. This DNA can persist and potentially be detected for hours to weeks, depending on environmental conditions (Joseph et al., 2022). As a result, eDNA signals may reflect transported material rather than local presence, potentially leading to overestimation of beta diversity. Nearshore marine ecosystems such as eelgrass, kelp forests, and mudflats often occur within this 10‐km range of one another, facilitating exchange of detrital and drift material that can artificially inflate species richness at a site. Despite the large and conspicuous nature of many “off site” taxa, confidently distinguishing drift detections from locally established species remains impossible without complementary visual surveys.

Nevertheless, although metabarcoding introduced some biodiversity noise through drift detections, several groups were observed exclusively by eDNA, including cryptic, crustose, and ephemeral taxa groups for which dive collections are challenging or impractical. These taxa collectively accounted for 17.5% of all eDNA‐only species detections. Cryptic and crustose species were identified as significant factors driving compositional differences between metabarcoding and dive surveys.

The significant differences in species composition of eDNA and dive surveys observed in this study can also be explained by amplification biases that may skew eDNA results. Primer and template DNA mismatches, particularly in the 3′ end, can severely reduce primer affinity and hinder amplification (Stadhouders et al., 2010). Successful eDNA amplification also depends on the availability of DNA material, either from shed material or spore release during reproductive periods. Several taxa with eDNA detection accuracy below 50% were robust and resistant to fragmentation (e.g., *Ahnfeltia plicata*, *Euthora cristata*), whereas delicate, highly branched species were readily detected by eDNA when present during dive surveys (e.g., *Bonnemaisonia hamifera*). Additionally, the overabundance of DNA from other taxa (e.g., red tides) may suppress amplification of target species and obscure detection of rare taxa. Amplification rates are species‐ and primer‐specific and, likely, a complex combination of these biases, which is not easily resolved.

### Detections of target species

Stochastic processes that can lead to false negatives or false positives are a source of variation in eDNA detections. We observed frequent false negatives from both qPCR and metabarcoding, even for abundant turf species. Although some in vitro studies reported higher detection sensitivity from qPCR than metabarcoding (McColl‐Gausden et al., 2023), a comparison performed by Harper et al. (2018) found similar sensitivity between the two methods once species detection thresholds were removed from the metabarcoding pipeline. Consistent with this, our study detected no difference in target species detection rates between qPCR and metabarcoding.

Rates of false negatives were high for both eDNA methods and may have been caused by PCR inhibition of non‐target DNA (Harper et al., 2018). Although increasing sampling efforts might seem like a solution to mitigate false negatives, Shirazi et al. (2021) demonstrated that even 21 eDNA samples were insufficient to eliminate false negatives. Moreover, higher sampling rates can increase false positive rates (Ficetola et al., 2015), often due to contamination or index hopping (van der Valk et al., 2020). To address these challenges, Shaw‐McDonald et al. (2025) recommended using a new classification framework for interpreting qPCR detections. Under this framework, a species is considered detected only if repeatable DNA detection occurs at either the qPCR replicate or site level (e.g., 100% of qPCR replicates within a sample or >1 sample replicate within a site; Shaw‐McDonald et al., 2025). Species presence is considered inconclusive if non‐repeatable DNA detection has occurred at both levels (e.g., <100% of qPCR replicates within a sample and detection in only 1 sample replicate within a site; Shaw‐McDonald et al., 2025). Given the potential for false negatives and false positives, eDNA detections should be treated as proxies for species presence and followed up by visual confirmation when possible (LeBlanc et al., 2020). For long‐term monitoring programs, scanning a broad region with eDNA methods and using key positive or negative detections to guide follow‐up surveys by traditional methods (e.g., diving) offers an efficient and reliable approach. For studies monitoring the spread of invasive species, we caution that new arrivals of invasive species or range expansions should not be reported based solely on eDNA and should always be confirmed through direct observation and identification.

### Limitations of the study

The use of eDNA has become commonplace among marine surveys; however, its application remains largely unstandardized. For metabarcoding, limited reference databases are a major constraint. In this study, only 19% of macroalgal sequences could be identified using available reference databases, while the remaining 81% lacked species‐level matches. Additional taxa were likely present in the eDNA samples but could not be identified due to missing reference sequences. As sequencing efforts expand and public databases improve, the proportion of useful metabarcoding data will increase. The metabarcoding approach in this study aimed to capture broad marine diversity, and the universal primers used were not specifically designed for macroalgae. We demonstrated that these primers, particularly the Leray fragment, performed reasonably well for macroalgae surveys in this region, but they may have hindered the detection of some taxa (e.g., most kelps and *Codium fragile*). Replacing or supplementing the 18S–V4 amplicon with plastid‐targeting marker (e.g., the *rbc*L gene) or adopting a targeted metabarcoding approach (see Wu et al., 2021) may improve detection when macroalgae are the primary focus. Given the substantial genetic divergence among brown, red, and green algae, primer sets may need to be designed for each phylum individually. However, greater specificity comes at the expense of taxonomic breadth and increases analytical costs. Further understanding of shedding rates for macroalgal species, given their differences from invertebrates and vertebrates, would enable more accurate timing for eDNA sampling.

## CONCLUSIONS

Using in situ sampling, we evaluated the high diversity and detection sensitivity of eDNA methods compared to traditional dive surveys. Although metabarcoding captured substantially higher diversity, some detections could not be attributed to locally established species, and eDNA sensitivity was reduced by frequent and unpredictable false negatives that may have overlooked ecologically significant taxa. Traditional sampling methods also have limitations: They are time‐intensive, require taxonomic expertise, and struggle to detect cryptic, crustose, and ephemeral taxa. For biodiversity applications, combining eDNA and traditional surveys provides the most comprehensive view and helps mitigate biases from primer affinity or seasonal patterns in eDNA. For monitoring invasive taxa or species distributions, eDNA enables rapid screening across broad regions; however, key presences or absences should always be validated through secondary surveys. Overall, our results indicate that eDNA is a powerful complement to traditional methods but should not replace them for characterizing macroalgal species presence and diversity.

## AUTHOR CONTRIBUTIONS

**Cody M. Brooks:** Conceptualization (equal); data curation (lead); formal analysis (lead); investigation (lead); methodology (equal); software (lead); visualization (lead); writing – original draft (lead). **Claudio DiBacco:** Conceptualization (equal); funding acquisition (equal); investigation (equal); methodology (equal); project administration (equal); resources (equal); supervision (equal); visualization (equal); writing – review and editing (equal). **Meghan C. McBride:** Investigation (equal); methodology (equal); writing – review and editing (equal). **Kira A. Krumhansl:** Conceptualization (equal); funding acquisition (equal); investigation (equal); methodology (equal); project administration (equal); supervision (equal); writing – review and editing (equal).

## Supporting information


**Table S1.** qPCR primers used to target six macroalgal species in this study. Gene targets are the mitochondrial cytochrome *c* oxidase I (COI), chloroplast elongation factor Tu gene (*tuf*A), and chloroplast ribulose‐1,5‐biphosphate carboxylase‐oxygenase (*rbc*L). Except for the assay for *Bonnemaisonia hamifera*, all qPCR assays were designed for this study.

**Table S2.** Metabarcoding primers used to target the Leray fragment of COI‐5P and V4 region of 18S in this study.

**Table S3.** Species abundances by site for all species observed in this study. Abundances from dive surveys are reported as mean biomass (g per 0.25 m^2^ dry weight) from eight (in 2021) or six (in 2022) quadrats. Abundances from eDNA are reported as combined Leray fragment and V4 read counts across all samples from a given site. Grouping factors used for Spearman's correlations are also provided for each species.

**Table S4.** Spearman correlations for all grouping factors assessed in this study. Spearman's ρ, *p*‐values and *S*‐statistics are provided for all correlations.
