# Supplementary material for: Dive surveys and eDNA detect contrasting macroalgal community composition in Nova Scotia, Canada
Source: J Phycol. 2026 Jul 23;62(5):1510–24. doi: 10.1111/jpy.70208 (PMC13659366; doi:10.1111/jpy.70208)
Supplement: Supplementary file 1 [file JPY-62-1510-s002.docx]

| Target | Primer Description | Primer Name | Primer Sequence (5`→3`) | Source |
| --- | --- | --- | --- | --- |
| LERAY | Forward Primer | mlCOIintF | GGWACWGGWTGAACWGTWTAYCCYCC | (Leray et al., 2013) |
|  | Reverse Primer | jgHCO2198 | TANACYTCNGGRTGNCCRAARAAYCA | (Geller et al., 2013) |
| V4 | Forward Primer | V4F | GCAGTTAAAAAGCTCGTAG | (Stat et al., 2017) |
|  | Reverse Primer | V4R | TCCAAGAATTRCACCTCT | (Stat et al., 2017) |

Geller, J., Meyer, C., Parker, M., & Hawk, H. (2013). Redesign of PCR primers for mitochondrial cytochrome c oxidase subunit I for marine invertebrates and application in all-taxa biotic surveys. *Molecular Ecologyl Resources*, *13*(5), 851-861. https://doi.org/10.1111/1755-0998.12138

Leray, M., Yang, J. Y., Meyer, C. P., Mills, S. C., Agudelo, N., Ranwez, V., Boehm, J. T., & Machida, R. J. (2013). A new versatile primer set targeting a short fragment of the mitochondrial COI region for metabarcoding metazoan diversity: Application for characterizing coral reef fish gut contents. *Frontiers in Zoology*, *10*, 34.

Stat, M., Huggett, M. J., Bernasconi, R., DiBattista, J. D., Berry, T. E., Newman, S. J., Harvey, E. S., & Bunce, M. (2017). Ecosystem biomonitoring with eDNA: metabarcoding across the tree of life in a tropical marine environment. *Scientific Reports*, *7*(1), 12240. https://doi.org/10.1038/s41598-017-12501-5
