# Supplementary material for: Dive surveys and eDNA detect contrasting macroalgal community composition in Nova Scotia, Canada
Source: J Phycol. 2026 Jul 23;62(5):1510–24. doi: 10.1111/jpy.70208 (PMC13659366; doi:10.1111/jpy.70208)
Supplement: Supplementary file 2 [file JPY-62-1510-s003.docx]

| Species | Target | | Primer Description | | Primer Sequence (5`→3`) | Source |  |
| --- | --- | --- | --- | --- | --- | --- | --- |
| *Antithamnion sparsum* | *rbc*L | Forward Primer | | TCAAGGGATCTTCTTCGAGCAA | |  | |
|  |  | Reverse Primer | | GGACCTTCTCCTACATAGTCAC | |  | |
|  |  | Probe | | QSY-GTGCAACAGCAAATCGTGTAGCTTTAGAAGC-JUN | |  | |
| *Bonnemaisonia hamifera* | *rbc*L | Forward Primer | | CAATTACTAGATTACCTGGGCAAT | | (Andersen et al., 2018) | |
|  |  | Reverse Primer | | CTTCTTTTACAAAGTCCCGACCT | |  |  |
|  |  | Probe | | FAM-TCGTGCCATAACCATAGACTCTAAAGCC-BHQ | |  |  |
| *Codium fragile* | *tuf*A | Forward Primer | | AATGGCTATTGAAAATGTTGTCTCAA | |  | |
|  |  | Reverse Primer | | CAACTGTCTGGCCAACTTCGA | |  | |
|  |  | Probe | | FAM-AAGAGGTACGGTTGCTAC-MGB-NFQ | |  | |
| *Dasysiphonia japonica* | COI | Forward Primer | | GGTAGCCCGGATATGGCTTT | |  | |
|  |  | Reverse Primer | | TTGAACTTAGCGGAGGATAAACTGT | |  | |
|  |  | Probe | | FAM-ATTATTGCCACCTTCACTTT-MGB-NFQ | |  | |
| *Laminaria digitata* | COI | Forward Primer | | TGCCTCCCTCTTTAATCCTTCTT | |  | |
|  |  | Reverse Primer | | CCCCGAGTGAGCCTGAATAC | |  | |
|  |  | Probe | | FAM-TCTACCCCCCCCTTAGT-MGB-NFQ | |  | |
| *Saccharina latissima* | COI | Forward Primer | | TTCAGGCACACTCAGGACCTT | |  | |
|  |  | Reverse Primer | | AGACAGACCATACAAAAAGAGGTAACC | |  | |
|  |  | Probe | | FAM-TTGACTTAGCTATATTTAGTCTCC-MGB-NFQ | |  | |

Andersen, J. H., Kallenbach, E., Thaulow, J., Hesselsoe, M., Bekkevold, D., Hansen, B. K., Jacobsen, L. M. W., Moller, P. R., & Knudsen, S. W. (2018). *Development of species-specific eDNA-based test systems for monitoring of non-indigenous species in Danish marine waters* (Report No. 7204-2017). Norwegian Institute for Water Research. <https://nva.sikt.no/registration/0198cc6370a1-7ff08e58-661b-45ef-ac39-a3f17cd6494e>
